# Grasping an object comfortably: orientation information is held in memory

**DOI:** 10.1007/s00221-015-4360-3

**Published:** 2015-07-01

**Authors:** K. Roche, R. Verheij, D. Voudouris, H. Chainay, J. B. J. Smeets

**Affiliations:** Laboratoire EMC, EA 3082, Université Lumière Lyon 2, Lyon, France; Department of Human Movement Sciences, MOVE Research Institute Amsterdam, VU University Amsterdam, Amsterdam, The Netherlands; Department of Psychology, Justus-Liebig University Giessen, Giessen, Germany

**Keywords:** Visuomotor behavior, Priming, Orientation, Grasping, Comfort

## Abstract

It has been shown that memorized information can influence real-time visuomotor control. For instance, a previously seen object (prime) influences grasping movements toward a target object. In this study, we examined how general the priming effect is: does it depend on the orientation of the target object and the similarity between the prime and the target? To do so, we examined whether priming effects occured for different orientations of the prime and the target objects and for primes that were either identical to the target object or only half of the target object. We found that for orientations of the target object that did not require an awkward grasp, the orientation of the prime could influence the initiation time and the final grip orientation. The priming effects on initiation time were only found when the whole target object was presented as prime, but not when only half of the target object was presented. The results suggest that a memory effect on real-time control is constrained by end-state comfort and by the relevance of the prime for the grasping movement, which might mean that the interactions between the ventral and dorsal pathways are task specific.

## Introduction

It has been proposed that visual information is processed through two pathways: one for perception and the other one for action (Goodale and Milner 1992). Originally, the vision-for-action pathway (or dorsal pathway) was proposed to process egocentric visual information in order to program and control goal-directed movements in real time independently from other processes. In contrast, the vision-for-perception pathway (or ventral pathway) was suggested to be in charge of perceptual and cognitive processes, such as identification and memory, taking into account the visual context. However, the independence of these two pathways was questioned as various interactions between them were reported (Rizzolatti and Matelli 2003; Gallese 2007). Thus, Milner and Goodale reviewed their visual pathways model including two major modifications: Ventral/dorsal interactions occur, and the dorsal pathway is not limited to the control of movements in real time (Milner and Goodale 2008). In spite of these modifications, it is still debated how visual information is processed in the two visual streams to guide goal-directed actions (Smeets et al. 2009; Goodale 2011). The general purpose of our study is to contribute to this debate.

The model of two visual systems (Milner and Goodale 2008) predicts that, under specific conditions, the programming and execution of an action are not only based on dorsal but also on ventral processes, especially if the action is unfamiliar and unpractised or if visual information is not available. Indeed, unpracticed actions are sensitive to contextual information. In particular, if the movement required to perform the task is awkward or unfamiliar, then automatic dorsal processes are not sufficient, and a more conscious control of the action is helpful (Gonzalez et al. 2008). The ventral pathway may also be involved if vision is not available during action execution (Milner et al. 1999).

The consequence of the assumed division of labor between the two streams is that grasping a visible target will not use any kind of memorized information about the target (Milner and Goodale 2008). Therefore, grasping a visible target will not be primed by information acquired earlier. However, more recent data suggest that this dichotomy between the two streams is not strict (e.g., Loh et al. 2010). Consequently, it is possible that dorsal and ventral processes interact in order to grasp an object when visual feedback is present (McIntosh and Lashley 2008; Schenk and McIntosh 2010). A possibility to study dorsoventral interactions is to use a visuomotor priming paradigm. Visuomotor priming consists of presenting a visual stimulus before the appearance of the target in order to influence the motor response to that target. It is a well-suited paradigm to study the potential influences of memorized information involved in a grasping task. Interestingly, some experimental data suggest that grasping is susceptible to visuomotor priming (Hesse et al. 2008; Masson et al. 2011; Roche and Chainay 2013). For instance, initiation times are shorter when a prime with the same orientation as the target is presented 100 ms before the target (Craighero et al. 1998), suggesting that a congruent orientation previously seen in prime leads to a facilitation of the visuomotor planning. This interpretation was questioned: As the targets in that study were not visible during the movement execution, the effect of the prime might have been on memory-guided movements and might not have involved any real-time visuomotor priming (Cant et al. 2005). Goodale and colleagues reported no orientation priming effects on action initiation time (Garofeanu et al. 2004; Cant et al. 2005) and therefore proposed that the dorsal pathway does not use visual information that was presented prior to the task (Goodale et al. 2006). However, a later study showed that priming effects are also clearly present when the target is continuously visible (Hesse et al. 2008). In particular, people are faster in initiating grasping movements to visible objects when the target is preceded by a prime with the same (congruent) orientation than by a prime with a different (incongruent) orientation. Thus, in general, it seems that it is possible to observe visuomotor priming effects.

The previously mentioned studies used abstract shapes (e.g., Craighero et al. 1998; Cant et al. 2005; Hesse et al. 2008) or pictures of everyday objects (Masson et al. 2011) as stimuli. Priming effects have also been found for three-dimensional tools under certain conditions. Visuomotor priming with everyday tools was observed only if the task required to point at it (Roche and Chainay 2014) or grasp it (Roche and Chainay 2013); it has been reported that this depends on the details of the task, as the priming was absent in a situation that did not involve the actual use of the object (Valyear et al. 2011).

All the above-mentioned studies used the initiation time of the grasping movement as a measure to examine the priming effects. The initiation time of grasping movements does not only result from visual and motor computations related to the target and the appropriate action, but also from the time needed for the decision to start the movement. According to the theory of the two visual systems, the ventral pathway is involved in the selection of action that is appropriate for the target, given the task (Milner and Goodale 2008). Taking this into account, the selection of the action and the target (ventral processes), as well as some primary computations of the upcoming hand movement (dorsal processes), takes place before the start of a movement. Therefore, the analysis of movement initiation time is relevant to study interactions between dorsal and ventral pathways (Schenk and McIntosh 2010). However, initiation time does not represent all the movement programming, because participants can start a movement before having processed all information needed for that movement (van Sonderen and Denier van der Gon 1991). We therefore analyzed also movement execution.

Measuring parameters describing movement execution is of added value because it is a way to probe the (dorsal) real-time computations that underlie the execution of visually guided actions (Masson et al. 2011). A parameter that is essential in grasping is the final grip orientation as it largely determines the stability of the grasp. Final grip orientation is a measure sensitive to the orientation of the target object (Cuijpers et al. 2004; Voudouris et al. 2012). Studies showing a stimulus–response compatibility effect support the idea that perception of a tool’s handle orientation may automatically activate motor components for grasping (Hesse et al. 2008; Roche and Chainay 2013). This effect shows that the orientation of stimuli automatically facilitates motor responses when the orientation is congruent, presumably by activating some motor components. If such activated motor components can be held in memory for a short time, orientation priming effects on the final grip orientation can be expected.

To examine the influence of the awkwardness of the grip and the similarity between the prime and the target object on this priming effect, we asked participants to grasp an elongated familiar object after viewing a prime. In all three experiments, we varied the orientation of both the prime and the target object, whereas in experiment 3, we also varied the similarity between the prime and the target object. If the remembered visual information from the prime is used, we should find priming effects on grasp parameters such as initiation time and final grip orientation. If information used to execute grasping is exclusively computed in real time, we should not find any priming effects. Examining which conditions lead to priming effects might result in more insight into whether and when interactions between the ventral and dorsal pathways occur.

## Experiment 1

### Methods

#### Participants

Eight participants (5 women and 3 men) from the Faculty of Human Movement Sciences of the VU University Amsterdam took part in this experiment. Their mean age was 27 ± 2 years (in this paper, we always present mean ± standard deviation). All were right-handed by self-report, with normal or corrected-to-normal vision. The participants were naive to the purpose of the experiment, and they signed an informed consent form before the experiment. The experiment was part of a program that has been approved by the ethics committee of the Faculty of Human Movement Sciences of the VU University.

#### Apparatus and stimuli

Movements of the thumb and index finger of the participants’ right hand were recorded using a two-camera Optotrak 3020 system (Northern Digital, Waterloo, ON, Canada) at a sampling rate of 200 Hz. Single infrared light-emitting diodes (IREDs) were attached to the nails of the thumb and index finger of the participants’ right hand using elastic gum.

**Fig. 1:**
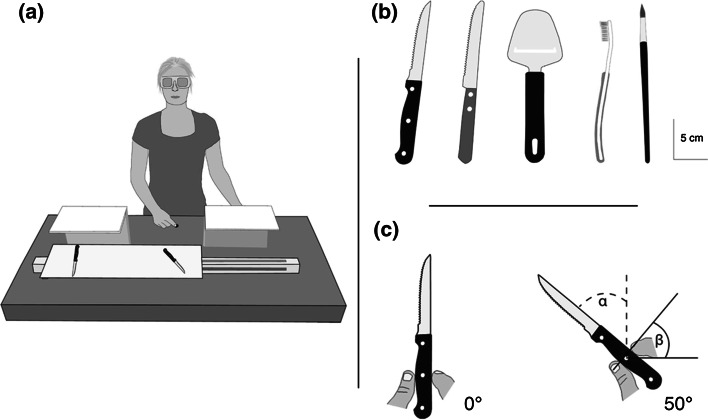
**a** Experimenter’s view of the setup. Two identical objects were placed on the sliding board, but their orientation could differ. The starting position is indicated by the *small black disk*. The participant could not see the object that was behind the side occluder. **b** Schematic drawing of the tools used in experiment 1 and 2. Note that participants interacted with real three-dimensional objects. **c** The orientations used in experiment 1. Note the different definitions of orientation of the target (*α*) and grip (*β*). In this way, an orthogonal grip corresponds to the same value for *α* and *β*

Participants sat in front of a height-adjustable table (122 × 60 cm), on top of which a sliding apparatus (60 × 20 cm) was placed. This apparatus consisted of a sliding board and two vertical occluders (Fig. 1a). Participants wore liquid–crystal shutter glasses (Plato Translucent Technologies, Toronto, Ont.), which could control participants’ vision by changing between being either opaque or transparent. Five handled tools were used, each as both prime and target (pencil, toothbrush, two kinds of knifes, and a cheese slicer; Fig. 1b). Each object was placed on the table in one of two orientations: 0° or 50° with respect to the participants’ sagittal axis (Fig. 1c). The starting position of the hand was at 31 cm distance from the stimulus’ middle and about 20 cm away from the participant’s trunk in the sagittal plane (Fig. 1a).

#### Procedure

The height of the table was adjusted so that each participant could sit comfortably. The experimenter made sure that the participant was not able to see behind the side occluders. While the PLATO glasses were opaque, the experimenter placed the prime at the visible area of the sliding board and the target behind one side occluder. A “beep” reminded the participant that soon (after 200 ms) the glasses would become transparent and that he or she would have to look and focus on the prime. The glasses then became transparent for 500 ms, before becoming opaque again for 1500 ms. The latter duration is less than two seconds, the time after which visuomotor information starts to decay (Hesse and Franz 2009). During this short period in which vision was removed, the experimenter moved the sliding board so that the target was brought at the exact location where the prime was seen before. The glasses then became again transparent for 3000 ms. During this time, the participant was to reach and grasp the target object with a precision grip (between thumb and index finger), lift it, place it back, and move his or her hand back to the starting position. The participant was instructed to initiate the grasping movement as fast as possible. No other instructions were given. Five practice trials were performed before the start of the actual experiment.

Each of the 20 conditions (2 congruencies between prime and target, 2 target orientations, 5 objects) was presented five times, resulting in 100 trials. The trials were presented in a pseudorandom order. In order to prevent any effect of target order, we divided the trials in two blocks of 50 trials each, and switched the order of presentation of these two blocks for half of the participants.

#### Data analysis

We determined the start of the movement as the moment at which the average position of the two digits’ markers exceeded a velocity threshold of 10 mm/s. The *initiation time* was defined as the time between the presentation of the target and the start of the movement. Trials with an initiation time of less than 100 ms or more than 1000 ms were excluded. The moment of the grasp (i.e., the first contact with the object) was defined as the last minimum of the averaged digits’ position in height, before this mean position reached its maximum height (which always occurred when lifting the object). The *final grip orientation* was determined as the orientation of the projection on the horizontal plane of a line connecting the markers on the thumb and index finger at the moment of the grasp. An angle of 90° between the thumb and index finger corresponds to both fingertips being along the sagittal axis of the participant (with the thumb closest to the body; Fig. 1c), with an anticlockwise rotation being positive. By using this definition of final grip orientation, an orthogonal grasp is achieved if this variable has the same value as the target orientation. We also calculated the orientation of the grip at the start of the movement (*start grip orientation*).

The values of the above-mentioned variables were calculated for each trial and then averaged across the five repetitions of each condition performed by each participant. The average standard deviations between the mean values of all participants and within the repetitions of each participant were also calculated. Since the experimenter reported difficulties to place the cheese slicer always at exactly the correct position, probably because it was the widest and the shortest object, we excluded the cheese slicer from all the analyses.

Preliminary analyses were conducted to check for normality (Shapiro–Wilk’s test) and sphericity (Mauchley’s test), with no violations found on all the experiments presented in this study. Effects on initiation time and final grip orientation were evaluated using repeated-measures ANOVA with factors target orientation (0° vs. 50°) and congruency (congruent vs. incongruent). Significance level was set to *p* < .05. The means and between-participant standard deviations for significant differences are mentioned in the results. We also conduct a *t* test on the *start grip orientation* between congruent and incongruent conditions.

### Results

**Fig. 2:**
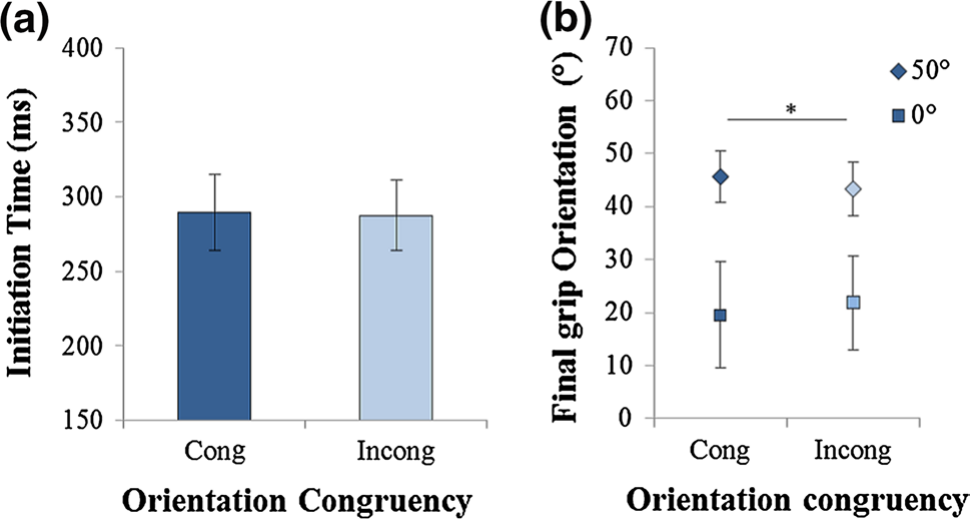
Experiment 1. **a** Mean initiation time for the two congruency conditions (Cong: congruent; Incong: incongruent); **b** Mean final grip orientation for the two congruency conditions and for the two target orientations (0° and 50°)

Based on the initiation times, approximately 4 % of the trials were excluded. Initiation time did not depend on congruency (*F*(1,7) = .06, *p* = .81, *η*^*2*^ = .008). Participants initiated the movement in the congruent condition as fast as in the incongruent condition (288 ± 24 ms; Fig. 2a). There was neither a significant effect of the target orientation on initiation time nor an interaction between congruency and target orientation (*p* = .9).

The final grip orientation was not influenced by congruency (*F*(1,7) < .0001, *p* = .99, *η*^*2*^ < .0001). Obviously, it was influenced by target orientation (F(1,7) = 20, *p* < .003, *η*^*2*^ = .74): The grip was oriented significantly more clockwise for the 0° target orientation (22° ± 7°) than for the 50° target orientation (44° ± 7°). There was also a significant interaction between congruency and target orientation (*F*(1,7) = 6.76, *p* < .04, *η*^*2*^ = .49; Fig. 2b). Planned comparisons showed that the interaction consisted of a significant congruency effect for the 50° condition *(p* < .04) but not for the 0° condition. More specifically, for the 50° target, the final grip orientation was slightly but significantly more counterclockwise in the congruent condition (45 ± 4°) compared with the incongruent condition (43 ± 5°).

The average start grip orientation was 64° (±4°) and was no different between the congruent and incongruent priming conditions (*p* = .78). The difference in grip angle between start and final grip orientation was smaller for the 50° target condition (64°–44° = 20°) than for the 0° target condition (64°–22° = 42°).

### Discussion

We examined whether visual information about the orientation of a prime is used in the programming and execution of a grasping movement when the target orientation is either 0° or 50°. This would be evident by observing effects on initiation time and on the final grip orientation. Surprisingly, no priming effects were found on initiation time. For final grip orientation, we found an interaction between congruency and target orientation: A priming effect on final grip orientation was observed when the target was oriented at 50°, but not when oriented at 0°.

That the priming effect on final grip orientation was only observed for a target at 50° might be explained by a difference in end-state comfort between the two target orientations (Rosenbaum et al. 1992, 1995; for a review, see Rosenbaum et al. 2006). The idea that comfort influences priming effects is compatible with priming effects (Masson et al. 2011). Masson et al. (2011) did not find priming effects when the target was not readily orientated in a proper way for the grasp and the use of the tool. The significance of the end posture is evident already in the programming procedure, as the motor cortex automatically activates stored action knowledge when a comfortable grip is associated with the orientation of the target object (Petit et al. 2006). Assuming that stored action knowledge is sufficient to induce priming effects (Tucker and Ellis 2004), memory of actions can be facilitated by a comfortable orientation. The programming and execution of an awkward grip may explain the lack of priming effects for targets in the 0° orientation of our experiment. An awkward grip requires more online visual feedback than a comfortable grip as the latter depends more on prior experience with the object and its current orientation (Norman 1999). Alternatively, the difference between the 0° and 50° target conditions might be explained by the difference in necessary rotations to bring the digits at the desired positions on the target object, because when grasping the target oriented at 0°, the difference between start grip orientation and final grip orientation is approximately twice as large compared with when grasping the target oriented at 50°, and planning a grasping movement takes into account the start posture of the hand (Kritikos et al. 1998; Hesse and Deubel 2009).

In contrast with previous studies (Craighero et al. 1998; Masson et al. 2011; Roche and Chainay 2013), we did not find any priming effects on initiation time. One can think that an experiment with two comfortable orientations could be more related to previous experience and action knowledge of the object and induce a priming effect on initiation time. To test this hypothesis and to better understand our results, we decided to replicate the first experiment but with different target orientations. In experiment 2, we used target orientations of 20° and 70°. In this way, we kept a difference of 50° between the two orientations, while aiming to avoid having a target that required the adoption of an awkward grip posture. We expected to find priming effects not only on final grip orientation but also on initiation time, for both target orientations.

## Experiment 2

### Methods

Eight participants (4 women and 4 men) from the Faculty of Human Movement Sciences of the VU University Amsterdam took part in this experiment. Their mean age was 27 ± 1 years. All were right-handed by self-report, with normal or corrected-to-normal vision. The participants were naive to the purpose of the experiment and signed an informed consent form prior to the experiment. Three of them had participated in experiment 1. The apparatus, stimuli, procedure, and analyses were the same as in experiment 1, except for the target orientations that were 20° and 70° (instead of 0° and 50°). The experiment was part of a program that has been approved by the ethics committee of the Faculty of Human Movement Sciences of the VU University.

### Results

**Fig. 3:**
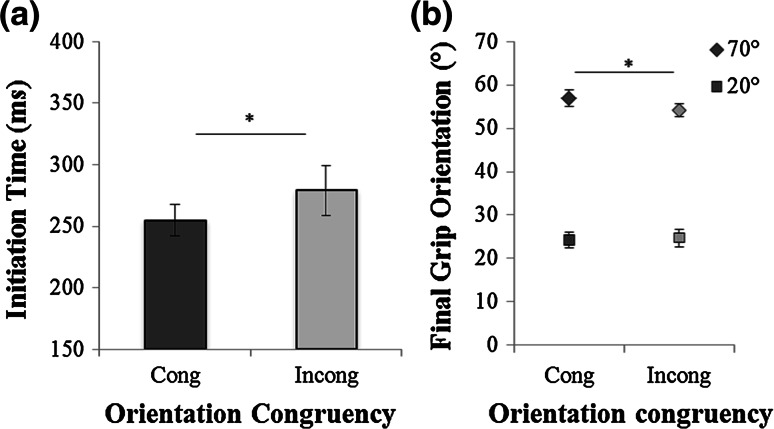
Experiment 2. **a** Mean initiation time for the two orientation congruency conditions (Cong: congruent; Incong: incongruent); **b** mean final grip orientation for the two orientation congruency conditions and for the two target orientations (20° and 70°)

Approximately 3 % of the trials were excluded. Initiation time was affected by congruency (*F*(1,7) = 5.6, *p* < .05, *η*^*2*^ = .44). Participants initiated their movements faster in the congruent condition (254 ± 12 ms) compared with the incongruent condition (279 ± 20 ms; Fig. 3a). There was no effect of target orientation on initiation time and no significant interaction between congruency and target orientation.

The final grip orientation was influenced by congruency (*F*(1,7) = 40, *p* < .01, *η*^*2*^ = .67): The grip was oriented slightly but significantly more counterclockwise in the congruent (40° ± 1.9°) than in the incongruent conditions (39° ± 1.7°). There was also a significant interaction between congruency and target orientation (*F*(1,7) = 5.96, *p* < .05, *η*^*2*^ = .46; Fig. 3b). Planned comparisons showed a priming effect for the 70° condition (*p* < .02) but not for the 20° condition. The 70° target was grasped systematically with more counterclockwise final grip orientations in the congruent (56° ± 3.6°) than in the incongruent condition (54° ± 2.4°). As expected, there was also a significant effect of target orientation on final grip orientation (*F*(1,7) = 613, *p* < .001, *η*^*2*^ = .98). The average final grip orientation was 24° ± 4° for the 20° target orientation and 55° ± 4° for the 70° target orientation. The average start grip orientation was 61° (±2°) and did not differ between congruent and incongruent priming conditions (*p* = .29).

To gain a better understanding in the differences in congruency effects on initiation times between experiment 1 and 2, we performed an ANOVA with the two experiments as factor. The ANOVA showed a significant effect of experiment (*F*(1,14) = 13.21, *p* < .01, *η*^*2*^ = .48) and a significant interaction between experiment and congruency (*F*(1,14) = 4.75, *p* < .05, *η*^*2*^ = .25). The analysis showed no significant effect of congruency (*F*(1,14) = 3.42, *p* = .08, *η*^*2*^ = .19). Planned comparisons of the interaction between *experiment* and *congruency* showed that initiation times were shorter in the congruent than incongruent condition in experiment 2 (*p* < .02) but not in experiment 1, in line with the results of the individual experiments. In addition, initiation time was longer in the congruent condition of experiment 2 than of experiment 1 (*p* < .001), but this difference was not significant for the incongruent condition.

### Discussion

In experiment 2, initiation time was influenced by the prime. Consistent with previous findings (Craighero et al. 1998; Hesse et al. 2008; Masson et al. 2011), seeing a congruent prime led to shorter initiation times. The fact that we did not find such an effect in experiment 1 could be attributed to the awkward grip required in that experiment. One can argue that a 20° prime is comfortable enough to invoke automatically stored action knowledge about tools and to facilitate movement initiation, but not comfortable enough to provoke an effect during movement execution, which probably should take into account constraints of the movement execution more precisely (Whür and Eslner 2007) and therefore influence the final grip orientation. Indeed, it seems that initiation and execution of the movement involved different constraints relative to their specialized processes (Glover 2004; Milner and Goodale 2008).

As in experiment 1, this present experiment showed that the priming effect on final grip orientation occurred only for the condition in which the target orientation was largest (70°). The choice of stimuli orientation at 20° was thought as a comfortable orientation compared to 0°. However, if we follow our explanation in terms of grip comfort, the lack of priming effect might suggest that 20° was not yet comfortable enough to evoke priming effects on final grip orientation. Taken together, the experiments 1 and 2 showed that the final grip orientation was sensitive to an orientation change that occurred prior the planning and programming of the movement and thus only if the movement did not require an awkward grip (Masson et al. 2011). We propose that the more comfortable a movement’s end posture is, the less the online adjustments are required, and the more the movement relies on memory of the prime.

Milner and Goodale (2008) claimed that, for unfamiliar movements, ventral processes interact with dorsal processes. If so, visuomotor priming effects would be present only for awkward movements and not for familiar and well-practiced movements. Our results are not in line with this prediction; hence, we believe that such a ventro-dorsal interaction is not underlying our priming effects. Our results are better explained by perceptual processes that interact with visuomotor processes (Gallese 2007; Schenk and McIntosh 2010), depending on the feasibility to adopt a comfortable grip posture (Masson et al. 2011).

## Experiment 3

In experiments 1 and 2, we found some priming effects induced by visual information. In experiment 3, we examined what visual information is necessary for priming effects to occur. Some studies have shown priming effects only when the prime was identical to the target, but not when they were different objects (Roche and Chainay 2013). Furthermore, motor facilitation occurs only when the prime is presented in a ready-to-use fashion that is linked closer to the history and knowledge about prior experience with the tool (Masson et al. 2011). In this third experiment, the prime was either the full object (a knife) or part of it (either the handle only or the blade only). If prior experience with tools and the associated action knowledge are involved in priming effects, then we expect that priming effects will only be found when the whole object is presented as prime, and not when only the handle or only the blade is presented.

### Methods

Eight participants (6 women and 2 men) from the Faculty of Human Movement Sciences of the VU University Amsterdam gave their informed consent and took part in the experiment. Their mean age was 28 ± 2 years. All were right-handed by self-report, with normal or corrected-to-normal vision. The participants were naive to the purpose of the experiment. Two of them had participated in experiment 2. The experiment was part of a program that has been approved by the ethics committee of the Faculty of Human Movement Sciences of the VU University.

**Fig. 4:**
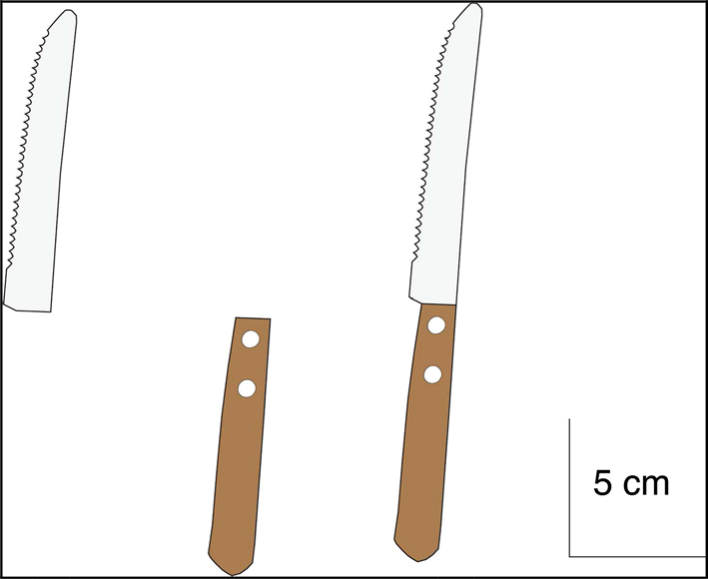
Schematic drawing of the object parts used in experiment 3. All three parts (*blade*, *handle*, *whole* object) were used as primes, but only the *whole* object was presented as a target

The apparatus, stimuli, and procedure were identical to those of experiment 2, except that we only used one object (knife) that was also used in the previous experiments as target. As prime we used either this whole object or a part of it: the handle or the blade (Fig. 4). The experiment consisted of 60 trials, combining three parts that could act as a prime (blade, handle, whole object) with two congruencies (congruent and incongruent) and two target orientations (20° and 70°). Each condition was presented five times. The trials were presented in a pseudorandom order. In order to prevent any effect of target order, we divided the trials in two blocks of 30 trials each and switched the order of presentation of these two blocks for half of the participants. A 3 (part) x 2 (congruency) x 2 (target orientation) repeated-measures ANOVA was performed on initiation time and final grip orientation.

### Results

**Fig. 5:**
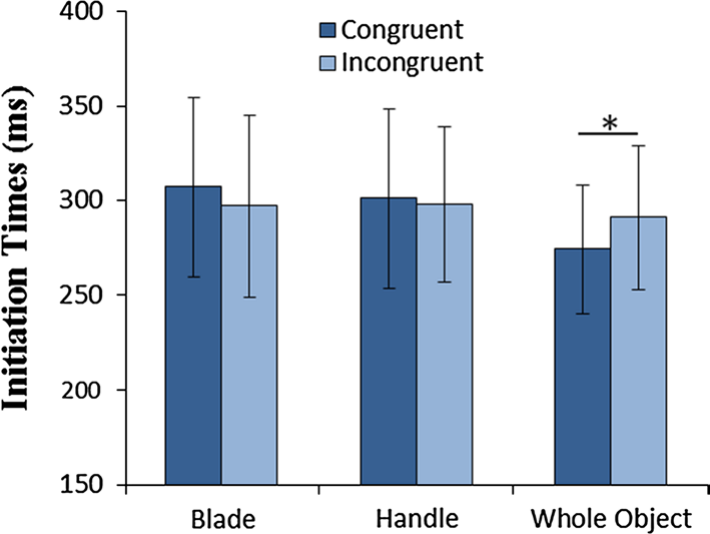
Experiment 3. Mean initiation time for the three priming conditions (*blade*, *handle* and *whole* object) as a function of congruency (congruent and incongruent). *Error bars* correspond to between—participants standard deviations

Approximately the 3 % of the trials were excluded from the data set due to initiation times being less than 100 ms or more than 1000 ms). The initiation time was affected neither by part (*p* = .5) nor by congruency (*p* = .07) nor by target orientation (*p* = .7). There was a significant part by congruency interaction (*F*(2,14) < 3.8, *p* < .05, *η*^*2*^ = .35). The planned comparisons showed shorter initiation time for the congruent (257 ± 33 ms) compared with the incongruent prime (295 ± 47), but only when the prime was the whole object (*p* < .05) and not when the handle (*p* = .6) or the blade (*p* = .3) were presented (Fig. 5).

Final grip orientation was neither affected by part (*p* = .7) nor by congruency (*p* = .6). Obviously, it was affected by target orientation (*F*(1,7) = 21608, *p* < .0001, *η*^*2*^ = .99): Final grip orientation was 30° ± 7° for the 20° target orientation and 60° ± 7° for the 70° target orientation. There was no significant interaction.

### Discussion

In experiment 3, we replicated the priming effects of experiment 2 on initiation time: Seeing the whole target in prime with the same orientation induces a shorter initiation time. Seeing only the blade or the handle is apparently not enough to induce a priming effect on initiation time. An orientation seen before with only a part of the target, with or without visual information relevant to the digits’ final position (the handle and the blade, respectively), does not speed up movement planning.

Symes et al. (2007) suggested that the object orientation is not the sole determinant of priming effects. Our results with the object’s parts in prime are consistent with this suggestion. To explain that the priming effect on initiation time occurred only when the prime was identical to the target and not when the prime was part of the target, we speculate that seeing the target in advance activates motor components that are used to plan the grasping movement because the whole object in prime is relevant to the grasping task. In this third experiment, the target was known in advance (it was always the whole knife). Based on our results, we can consider the prime as relevant when it was identical to the target (Allport 1987). Only a relevant prime leads to a facilitator effect. Such a viewpoint could be generalized to information selection in memory and thus explain that only relevant information is retained and therefore affects a subsequent action like grasping (Jax and Buxbaum 2010; Roche and Chainay 2013). In addition, it has been suggested that the detection of affordances of an object is influenced by the object’s function (Masson et al. 2011) and the actor’s capabilities (Ranganathan et al. 2011). This can also explain why we did not find an orientation priming effect on initiation time for the condition where object’s parts were presented as prime, because handle and blade primes were never presented as target, and thus they were never grasped.

We did not find any priming effect on final grip orientation in experiment 3, while in experiment 2 with the same orientations (20° and 70°), we found a priming effect for the 70° target. In experiment 2, five objects were used in two different orientations, whereas in experiment 3, only one object was used in two orientations. The number of different grasps was ten in experiment 2 compared with two in experiment 3. It is obviously easier for the participants to exactly replicate a grasping movement when only two, instead of ten, different grasps are required in the experiment. We can relate this idea to the tendency of dorsal processes to consider the recent action history (Whitwell and Goodale 2009) and to the notion of perseveration, which is a tendency to replicate a previously performed action (Dixon and Glover 2009; Glover and Dixon 2013). People may thus be more likely to replicate a previous performed action in experiment 3 than in experiment 2, which might lead to a lower sensitivity to priming effects in experiment 3 than in experiment 2.

## General discussion

In our three experiments, we found independent priming effects for initiation time (experiments 2 and 3) and final grip orientation (experiments 1 and 2). Our results suggest that initiation time can be reduced if one sees a prime identical to the target (experiment 3) in the same orientation as the target (experiments 2 and 3). This priming effect depends on the target orientation (experiment 1), presumably because the importance of online motor adjustments determines whether a priming effect will occur or not. Next to this, priming effects on final grip orientation seem to depend on the likelihood of priming conditions and/or prior actions leading to a successful grasp (based on the difference in results between experiments 1 and 2 and experiment 3). To summarize, if a prime affects the movement toward a target, the prime can influence both the initiation time and the final grip orientation but it can also influence only one of these variables. That the prime can influence only one of these variables could be explained by the fact that people can start a movement without finishing all the necessary processes related to the final orientation of the grip to correctly execute the movement (Whür and Eslner 2007).

Overall, our priming effects on both initiation time and final grip orientation imply a memory for visuomotor information of more than 1500 ms, which is much longer than the few hundred milliseconds of maximum memory proposed by Jax and Rosenbaum (2009) and Masson et al. (2011), but is consistent with the 2000-ms typical decay time of memory as suggested by Hesse and Franz (2009).

Another point of interest emerging from our three experiments is that the priming effects on initiation time and final grip orientation may depend on the comfort of the final posture. We speculate that these effects result from an interaction between real-time adjustments and prime treatments related to action knowledge coming from prior experience and the current orientation of a tool. We suspect that grasping an object with an awkward orientation demands more real-time adjustments of the hand’s orientation, which increases the importance of real-time visual feedback and, as a consequence, decreases the relevance of memorized information. Depending on the situation, more or less importance is given to the real-time processes. Because the dorsal pathway is assumed to only subserve online sensorimotor processes, we suggest that the memory capacity involved in our priming effects comes from an interaction between dorsal and ventral processes depending on the situation (Hesse and Franz 2009; Schenk and McIntosh 2010).

The influence of memorized information on real-time motor processes also raises the question of whether the memory capacity comes from the dorsal pathway or from ventral processes that interact with the dorsal processes. Our study does not provide a direct answer to this question. In spite of this, we found priming effects that were affected by end-state comfort, the kind of prime and the situation.

It has been shown that the intraparietal area and the posterior parietal cortex within the dorsal pathway are involved in the dynamical correction of grip orientation difference between the planned action and its execution (Desmurget et al. 1999; Tunik et al. 2005). Consequently, it seems that the dorsal pathway can use both memorized action plan and real-time information in order to efficiently execute a grasp. Furthermore, visual information that is processed within the ventral stream is also involved in a delayed grasping action (Cohen et al. 2009; Singhal et al. 2013). Taking these together, it seems that both ventral and dorsal pathways are involved during the execution of a grasping movement: the ventral pathway giving the memorized information to the dorsal pathway, which compares this information to the present situation and corrects the movement if necessary.

In sum, we found that viewing a tool in advance can affect initiation time and the final grip orientation of a grasping movement toward the tool. This priming effect was influenced by target object orientation, possibly because when the required grip is uncomfortable, priming effects are absent or masked because people need to rely more on online visual feedback. We suggest that the more relevant the online feedback is, the more subtle the priming effects are. The priming effect was also influenced by the similarity between the prime and the target. A priming effect occured when the prime and target object were identical but not when the prime was only a part of the target object. Together these results might mean that the interactions between the ventral and dorsal pathways are task specific.
